# CTCs as a prognostic and predictive biomarker for stage II/III Colon Cancer: a companion study to the PePiTA trial

**DOI:** 10.1186/s12885-019-5528-1

**Published:** 2019-04-03

**Authors:** Françoise Rothé, Marion Maetens, Ghizlane Rouas, Marianne Paesmans, Marc Van den Eynde, Jean-Luc Van Laethem, Philippe Vergauwe, Guido Deboever, Yacine Bareche, Caroline Vandeputte, Michail Ignatiadis, Alain Hendlisz

**Affiliations:** 1 J.-C. Heuson Breast Cancer Translational Research Laboratory, Institut Jules Bordet, Université Libre de Bruxelles, Brussels, Belgium; 2 Data centre, Institut Jules Bordet, Université Libre de Bruxelles, Brussels, Belgium; 3 Department of Medical Oncology, Cliniques Universitaires Saint-Luc, Université Catholique de Louvain, Brussels, Belgium; 4 Department of Gastroenterology, Erasme University Hospital, Brussels, Belgium; 5 Department of Gastroenterology, General Hospital Groeninge, Kortrijk, Belgium; 6 Department of Gastroenterology, Digestive Oncology, AZ Damiaan Ziekenhuis, Oostende, Belgium; 7 Gastrointestinal Translational Research Laboratory, Institut Jules Bordet, Université Libre de Bruxelles, Brussels, Belgium; 8 Medical Oncology Department, Institut Jules Bordet, Université Libre de Bruxelles, Brussels, Belgium

## Abstract

**Background:**

Adjuvant therapy improves the prognosis of stage II & III colon cancer patients. Unfortunately, most patients do not benefit from this treatment. PePITA (NCT00994864) is a prospective, multicenter, non-randomized study whose primary objective is to predict the outcome of adjuvant therapy in colon cancer.

**Methods:**

The primary objective was to determine the prognostic and predictive value of circulating tumor cell (CTC) detection before therapy and after one course of preoperative FOLFOX.

**Results:**

Out of the 58 first patients accrued in PePiTA trial, 36 patients participated in the CTC companion study, of whom 32 had at least one evaluable sample. Only 5 patients (14, 95% CI = 5–30%) had ≥1 CTC/22.5 ml blood in at least one of the two timepoints with 2 patients having ≥1 CTC/22.5 ml at baseline (6, 95% CI: 1–19%). The detection rate of patients with CTCs at baseline being lower than expected, the inclusion of patients in the PePiTA CTC substudy was stopped. The limited sample size did not allow us to investigate the prognostic and predictive value of CTCs in locally advanced colon cancer.

**Conclusions:**

Our data illustrate the need for further standardized studies in order to find the most reliable prognostic/predictive biomarker in early-stage colon cancer.

**Trial registration:**

This trial was prospectively registered at Jules Bordet institute (NCT00994864) on the October 14, 2009.

## Background

Despite slow but steady progress over the past decades, colorectal cancer remains a huge burden in terms of morbidity and mortality [1]. Currently, surgery is the only potentially curative modality for patients with localized colon cancer despite a significant recurrence rate, particularly in case of lymph node involvement [2]. In stage III colon cancer, FOLFOX adjuvant chemotherapy is associated with a statistically significant improvement in disease-free survival (DFS) and overall survival (OS) by more than 20% compared to surgery alone [3]. However, as long as no accurate predictive biomarker is available, most of the patients will draw little or no benefit from the adjuvant therapy.

Circulating tumor cells (CTCs) have been detected with variable levels in the peripheral blood of patients having advanced cancer from different origins [4, 5]. Changes in CTC count between baseline and the second anti-cancer treatment course in advanced breast cancer [6–8] have been associated with an adverse prognostic and predictive value on the patient’s outcome, with a reported good “negative predictive value of CTCs”. This finding has been reiterated in other metastatic cancer types, such as colorectal [9–17], prostate [18–20], melanoma [21, 22], gastric [23], ovarian [24] and lung cancer [25, 26].

The prognostic and predictive potential of CTC detection has also been explored in an earlier disease setting. While more than 1 CTC could be present in 15% of patients with benign condition or in 19% of “in situ breast carcinoma”, their presence is demonstrated in 19–27% of malignant breast cancer patients and is associated with early recurrence and decreased OS [27–29]. Uen et al. have studied 438 locally advanced colon cancer patients and collected blood samples for RNA extraction, using a membrane array method for the detection of CTC-related mRNA molecular markers 1 day before and 1 week after curative surgery. The results demonstrated the presence of circulating tumoral RNA in 31% of the patients and showed that this presence is an independent predictor for disease recurrence after curative surgery for CRC [30]. This finding has been confirmed in 5 (out of 8) other studies involved in a comprehensive literature review published in 2010 [31] and more recently in a study by Lu and colleagues [32]. All these studies used various time points for blood sampling and different RT-PCR techniques or membrane-array method in order to identify CTCs. However, there is very limited data concerning their use in the preoperative colon cancer setting to predict clinical outcome and to monitor therapeutic response to one course of chemotherapy given prior to surgery.

The PePiTA trial [33] is a prospective, multicenter, non-randomised study whose primary objective is to determine the predictive value of FDG-PET/CT-assessed tumoral metabolic changes after one course of preoperative FOLFOX on the outcome of adjuvant therapy in colon cancer as measured by 3-year disease-free survival. Here, we report the results of the exploratory CTC companion study aiming to assess the predictive value of the changes in CTC count after one course of preoperative FOLFOX and the prognostic value of the CTC count before any therapeutic intervention (baseline) in locally advanced colon cancer.

## Methods

### Study design and sample collection

**Fig. 1:**
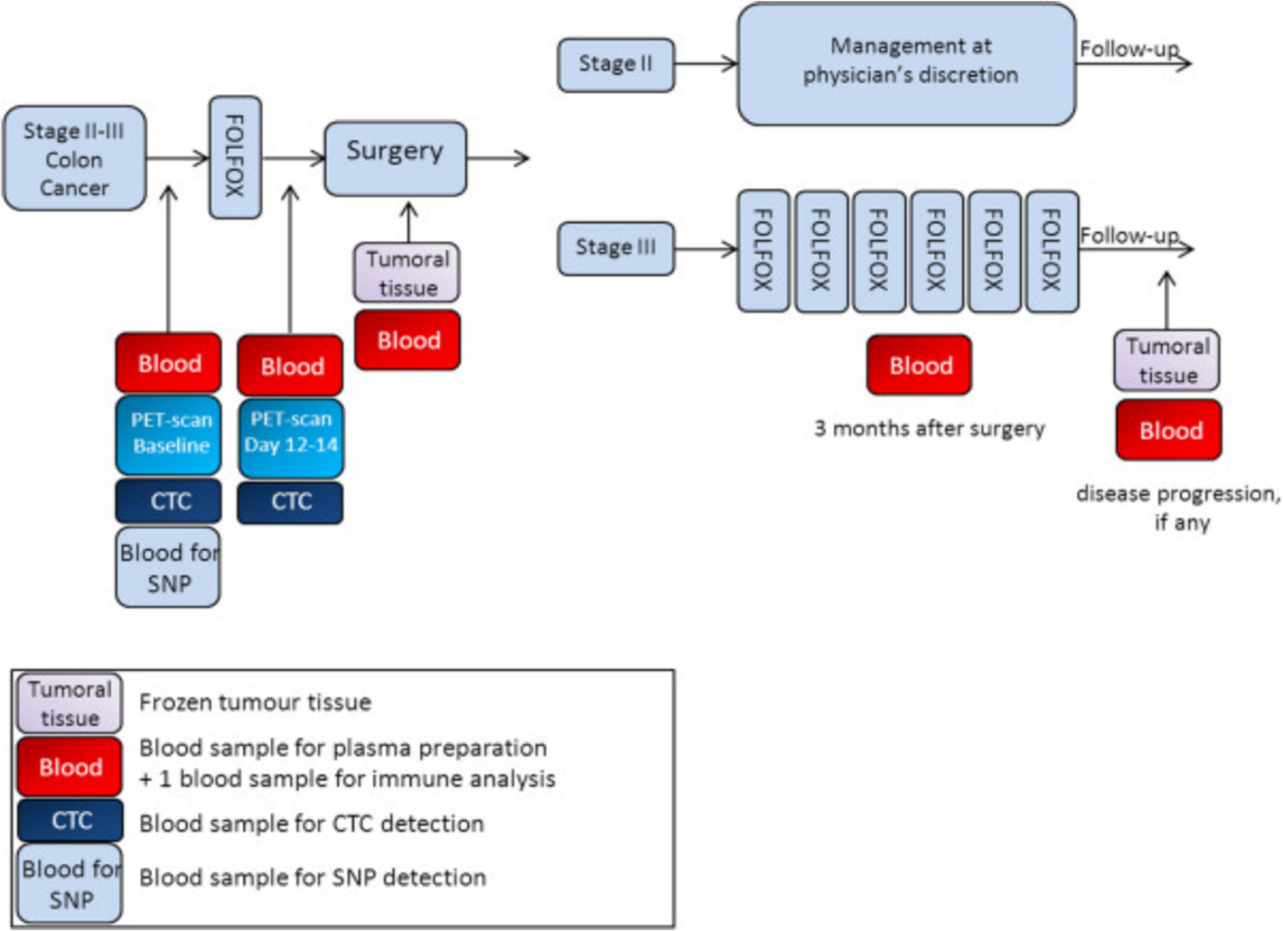
PePiTA study design

PePiTA (EudraCT number 2009–011445-13, ClinicalTrials.gov number NCT00994864) is a prospective, multicenter, non-randomized trial built on the hypothesis that preoperative chemosensitivity testing using FDG-PET/CT before and after one course of FOLFOX can identify the patients that are unlikely to benefit from 6 months of adjuvant FOLFOX treatment for stage III colon cancer. The trial design (Fig. 1), eligibility criteria and statistical analysis have previously been described [33]. The PePiTA trial is still accruing. The CTC companion study was optional. Patients gave their consent prior to study entry and before any study procedure. Blood samples for CTC analysis were prospectively collected before any therapeutic intervention (baseline) and after one course of preoperative FOLFOX (week 2). Each sample was shipped within 48 h to the central facility laboratory and processed for CTC analysis. The PePiTA companion study was performed in full accordance with relevant guidelines and regulations. Approval for the experimental protocol was obtained from the ethical committees from the Jules Bordet Institute, the Erasme University Hospital, the General Hospital Groeninge and the AZ Damiaan Ziekenhuis hospital. Written informed consent was retrieved from all participants.

### CTC detection

For each time point, 3 × 7.5 ml (22.5 ml in total) of peripheral blood was collected in CellSave preservative tubes and sent at room temperature to the Institut Jules Bordet (Brussels, Belgium) for central CTC analysis. The CTC enumeration was performed within 72 h after blood draw using the FDA-cleared CellSearch® system (Veridex, Raritan, NJ, USA) according to the manufacturer’s instructions. In order to avoid potential contamination from skin epithelial cells, the first 5 ml of blood draw were discarded from analysis [32]. The epithelial cells are captured using ferrofluid nanoparticles coated with antibodies targeting the Epithelial Cell Adhesion Molecule (EpCAM). The enriched sample is then stained with fluorescently labelled antibodies directed against cytokeratins 8, 18 and 19 (CK) and against CD45 and the nuclear dye 4′,6-diamidino-2-phenylindole (DAPI). HER2 expression on CTCs was characterized using the CellSearch Tumor Phenotyping reagent HER2/neu. CTCs were defined as epithelial, nucleated cells expressing cytokeratin but not CD45. CTC enumeration was performed using the CellTrack® Analyzer II, a semi-automated fluorescence-based microscope. Quality control regarding reagents and instrument standardization and operator technique was assessed using the CellSearch® CTC control kit. HER2 expression on CTCs was scored using a semi-quantitative method (0, 1+, 2+ and 3+) as previously described [34], HER2 positivity being defined as a HER2 staining intensity equal to 2+ and 3+. All samples were evaluated by two trained independent readers.

### Statistical analyses

Due to the exploratory nature of the study, we had no formal statistical hypothesis leading to a sample size estimation but the objectives of this companion study were to assess the prognostic value of baseline CTCs and to investigate the possible role of a decrease in CTCs between baseline and surgery. During an interim analysis carried out to assess the safety of the therapeutic plan and to monitor the study, we assessed the rate of patients in whom we were able to document a decrease in CTCs as well as the baseline detection rate. The observed proportions are provided together with 95% confidence intervals calculated through exact binomial distributions. The closure of the CTC companion study was decided because CTCs detection rate at baseline was very low implying virtually no chance to obtain a decrease in the CTC count following one course of chemotherapy. Additionally, we came to realize that the baseline detection rate was not high enough to allow any prognostic analyses to be run.

## Results

Out of 58 patients registered in the PePiTA trial at the time of the evaluation of the CTC companion study, blood samples for CTC analysis were obtained from 36 (62%) patients enrolled in 11 recruiting clinical sites. The reasons for absence of blood sample were: 1) no informed consent, 2) ineligibility and 3) blood draw not performed. The clinical characteristics of the 36 patients included in the CTC companion study of PePITA trial and the 17 eligible patients recruited in the study during the same period who did not participate to this companion study are reported in the Table 1. No differences in terms of age, pathological stage, tumor size or lymph node status could be observed between patients included or not in this sub-study.

**Table 1:** Patients and tumors’ characteristics from the PePiTA trial and CTC sub-study

	All patients	Patients in CTC substudy
Variable		
Sample size No.	53	36
Age No. years		
Median	67	67
Range	43–81	43–81
Gender No. (%)		
Male	32 (60)	25 (69)
Female	21 (40)	11 (31)
Stage		
I/II	9 (17) / 13 (25)	4 (11)/9 (25)
III/IV	27 (51) / 4 (8)	20 (56)/ 3(8)
Tumor Size No. (%)		
T1	1 (2)	1 (3)
T2	11 (21)	5 (14)
T3-T4	41 (77)	30 (83)
Lymph node status No. (%)		
Negative	22 (42)	13 (36)
Positive	31 (58)	23 (64)

**Fig. 2:**
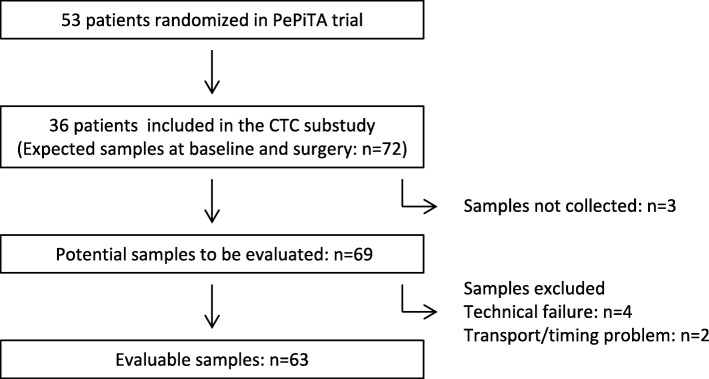
PePiTA CTCs sub-study flow chart

For the 36 included patients, a total of 69 blood samples were collected, of which 63 out of the 69 samples were evaluable. For 32 patients, 32 samples at baseline and 31 samples after one course of preoperative FOLFOX (Fig. 2) were valuable. Two samples were not assessed due to a timing error or transport problem and 4 were not assessable due to a technical failure. Out of the 32 patients with evaluable CTC, 5 (14, 95% CI: 5–30%) had ≥1 CTC/22.5 ml in at least one of the two time points (i.e. at baseline and after one course of preoperative FOLFOX) (Table 2). We detected ≥1 CTC/22.5 ml in 2 out of 32 (6, 95% CI: 1–21%) and 3 out of 31 (10, 95% CI: 2–26%) patients with evaluable samples at baseline and after one course of preoperative FOLFOX respectively. The patients positive at baseline were different from the patients that were positive for CTCs after one course of FOLFOX (Table 2). The association between the detection of CTCs and the clinico-pathological parameters could not be assessed because of limited sample size. However, as the 95% confidence interval for the rate of detection at baseline, among the patients included in the sub-study, was mostly below 20%, the CTC sub-study was stopped for futility and no more blood samples for CTC analysis were collected for patients enrolled in the PePiTA trial.

**Table 2:** CTC enumeration from patients included in the PePiTA sub-study

	No. of patients with detectable CTC at each time-point	
	Baseline	Pre-surgery
Patient ID		
1	1	0
2	0	2
3	0	1
4	0	1
5	1	0

Indeed, assuming a rate of 58% (31/53) of patients evaluable for CTCs out of the eligible patients, we could anticipate (with a planned sample size of 225 patients) a number of around 130 patients evaluable. Out of them, at most, 27 patients would be expected with baseline CTCs giving no chance to find any statistically and clinically significant prognostic value for a decrease in CTCs count. In those 130 patients, we could further estimate that 70% would be pathological stage II or stage III, i.e., 91 patients. The number of events was expected to be, at the end of the follow-up, 24. This means that, with the observed detection rate, we would have been able to detect prognostic value with hazard ratio in excess of 10. This appeared unrealistic and we closed the sub-study for futility. Of note, HER2 expression was evaluated on all CTCs (Table 2) and none of them were positive.

## Discussion

In clinical practice, stage III colon cancer patients are commonly treated by adjuvant chemotherapy after curative-intent surgery. However, the high recurrence rate (30%) and the evidence that almost 80% of the patients draw finally little or no benefit from adjuvant cytotoxic drugs highlights the need for predictive biomarkers.

In this companion study of the PePiTA trial [33], we found a CTC detection rate of 6% (2 out of 32 patients) preoperatively and 10% (3 out of 31 patients) after one course of chemotherapy, making an overall 14% (5 out of 32) in either of the time point. This rate is lower than originally anticipated from published data as in 5 out of 8 studies, a detection rate of 33.4% was observed [31] while all these studies used different techniques to detect CTCs. In the same line, Uen et al. detected CTCs in 82% stage II and III colon cancer patients (358 out of 438) preoperatively [30]. Van Dalum et al. have shown a 24% CTC detection rate in non-metastatic colorectal cancer patients before surgery using the CellSearch system [35]. Of note, recent meta-analyses including 20 studies with non-metastatic CRC revealed a detection rate ranging from 8.8 to 77% preoperatively with most studies using RT-PCR technology for CTC detection [36]. The association between the presence of preoperative CTCs and a recurrence-free survival and colon cancer related survival is controversial in the non-metastatic setting [36, 37]. Several reasons could explain the discrepancies in CTC detection rate between the different studies: (1) the different methodologies for CTC detection, (2) the differences in clinico-pathological characteristics of the patients and (3) the different time points of blood draw collection.

The CellSearch® system has been used for CTC detection in the present study. This is currently the only available FDA-approved technology for the detection and enumeration of CTCs. The system has been validated in the setting of metastatic breast, colorectal and prostate cancer. Other studies used RT-PCR technologies using a combination of different markers for CTC detection such as carcinoembryonic antigen (CEA), cytokeratin- 19 (CK19) and CK20 for CTC detection [31]. Several studies have already been published comparing different techniques for CTC detection. A direct comparison was made between the CellSearch® system, the AdnaTest involving immunomagnetic separation followed by RT-PCR and CK-19/mammaglobin RT-PCR in patients with metastatic breast cancer [38]. Different technologies were also compared for the detection of CTCs in CRC patients: a multimarker RT-PCR assay, the CellSearch® system and a dHPLC-based gene mutation analysis [39]. Interestingly, in both studies, the RT-PCR assays were significantly more sensitive than all other techniques used. Despite its higher sensitivity in CTC detection, an essential restriction of the RT-PCR assay is its inability to quantify the number of CTCs. The main disadvantage of the CellSearch® system is that it uses an EpCAM-based enrichment, a marker that might be downregulated in certain disseminated tumor cells [40]. Moreover, the CellSearch® system only takes intact cells into account, which might also explain the lower detection probability compared to RT-PCR based techniques. Another discrepancy between the different technologies is the amount of blood required. In our study, the expected low CTC detection in patients with early-stage colon cancer compared to metastatic patients was anticipated by collecting 3 times the volume of blood (22.5 ml) needed for the CellSearch® system. However, despite the large volume analysed, a very low detection rate was observed, suggesting that this technique may not be optimal in this setting.

As mentioned previously, the clinico-pathological characteristics also differ between the various studies on the detection of CTC in early-stage CRC. Most prominent are the differences in TNM stage varying from studies including only stage I or II patients to studies including patients with stages I to IV CRC [41–43] limiting a correct interpretation and comparison between the available data. The PePITA sub-study only included 2 stage II and 30 stage III patients. No statistical analysis on CTC detection between both stages could be performed due to the low overall rate of CTC positivity. Moreover, our study only included colon cancer patients while the other studies included patients with colon and with rectum cancer.

Finally, the time point of blood sample collection varies between studies. In this study, peripheral blood samples were collected pre-operatively both before and after one cycle of FOLFOX therapy. Different time points were evaluated in other studies, CTCs were detected peri-operatively, 24 h after tumor resection and even later than 48 h after resection [30, 41, 44]. Out of 53 eligible patients enrolled in the PePiTA trial, blood samples for CTC analysis were collected for 36 patients (68%) indicating low participation rate in translational research especially when the collection of biological material is optional, highlighting the difficulty to rely on optional sub-studies and questioning the need for minimizing the options in both ICFs and studies. Moreover, 63 out of 69 (91%) blood samples were evaluable due to transport and timing errors amongst others, increasing the failure rate.

## Conclusion

In conclusion, although most studies state that the presence of CTCs in peripheral blood is an independent factor of recurrence, it is clear that several factors need to be taken into account including time of blood sample collection, disease staging and a sample size high enough to allow proper statistical analysis. In our study, only 5 out of 36 patients were positive for the presence of CTCs, resulting in the finalization of CTC assessment in the PePITA sub-study. This very low detection rate did not allow to address the prognostic and predictive values of CTCs detection or change in the PePITA companion study. Moreover, it raises important questions regarding the appropriate technology for CTC detection in early-stage colon cancer. Further standardized studies are needed in order to find the most reliable biomarker for disease recurrence in early-stage CRC. It is important to note that only an accurate predictive biomarker or a combination of several markers can lead to suitable treatment decision excluding unnecessary chemotherapy-related toxicities.
